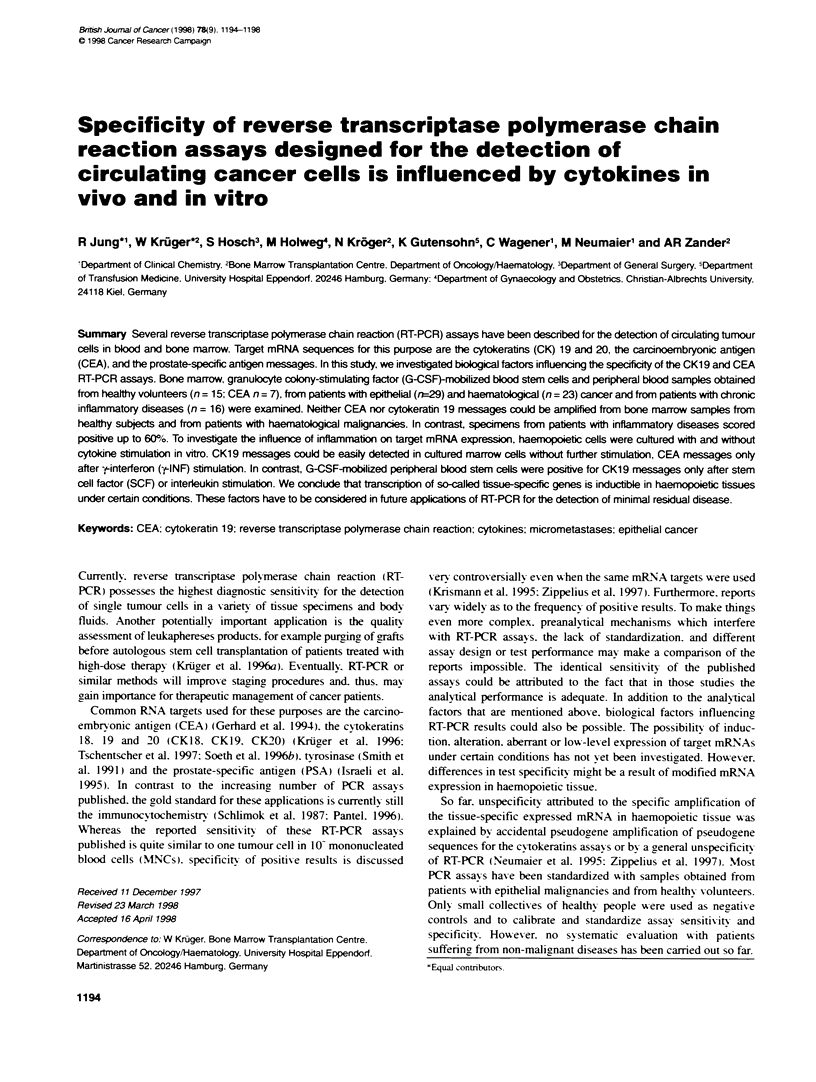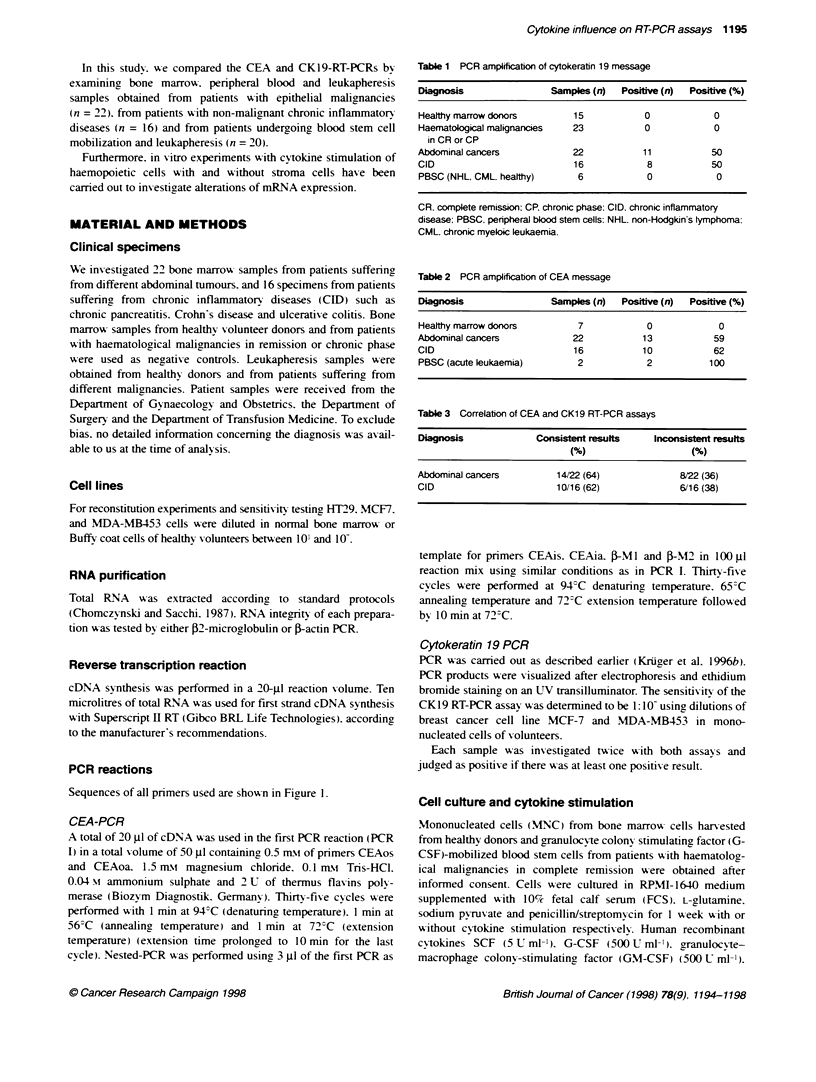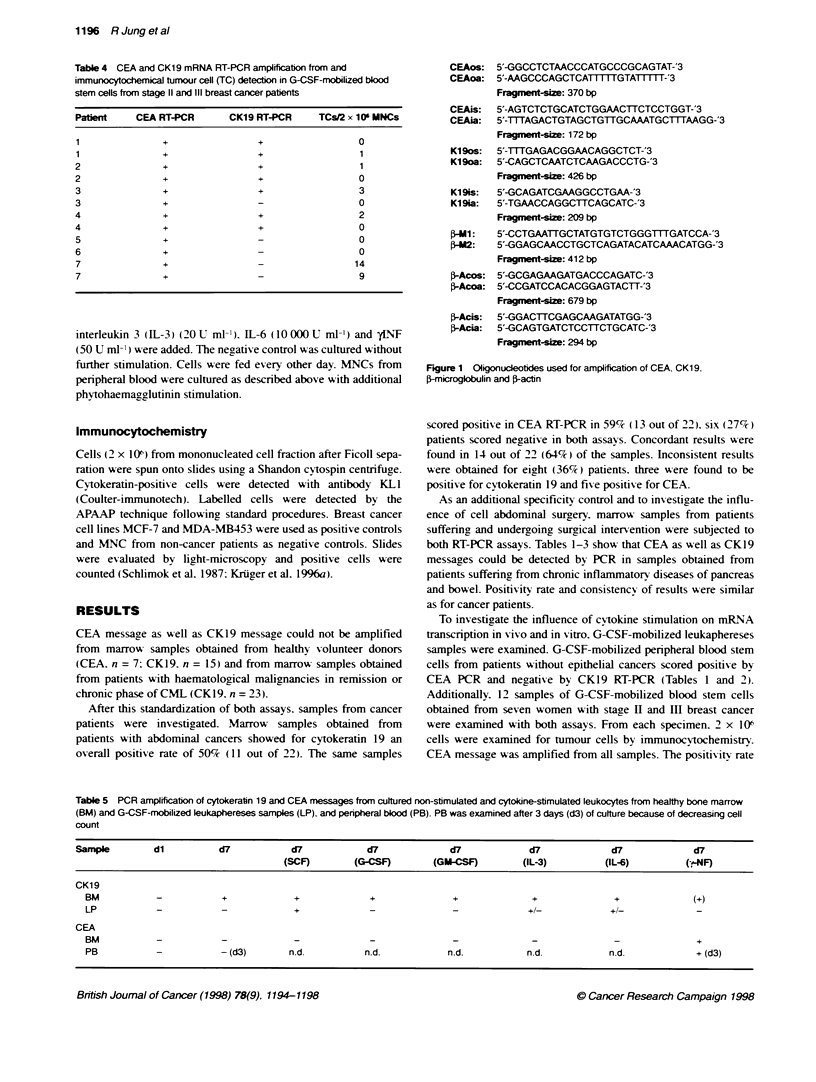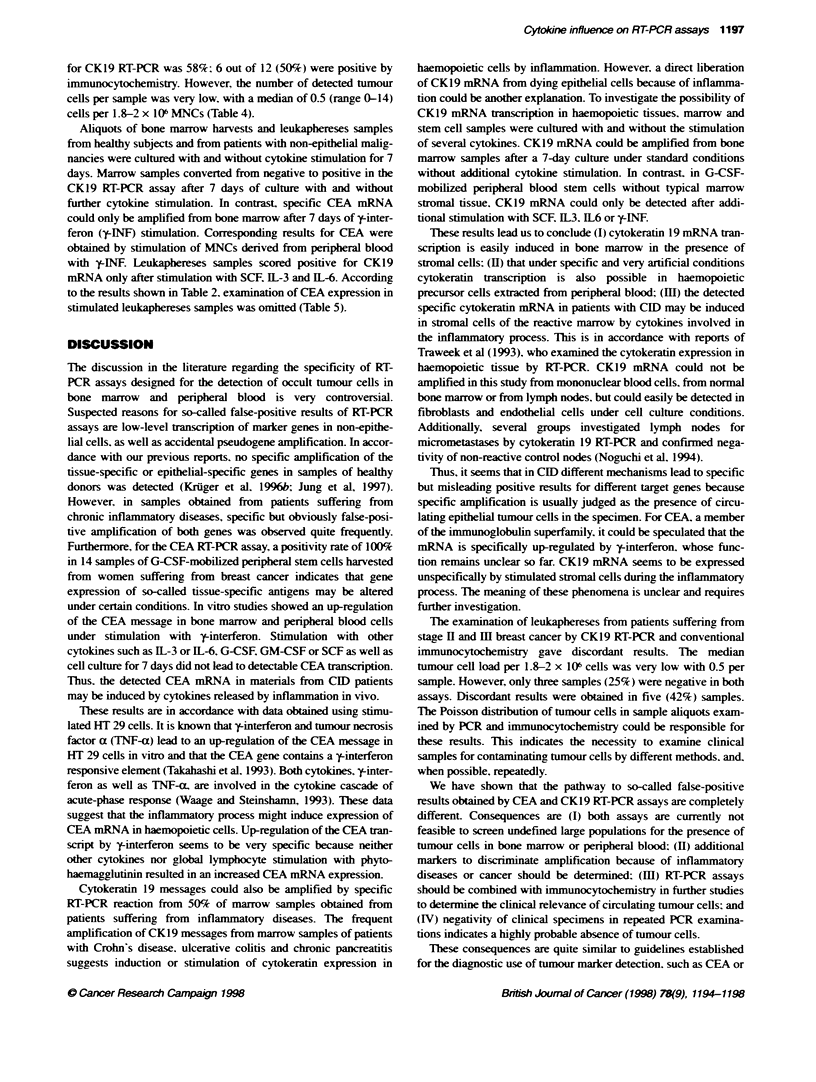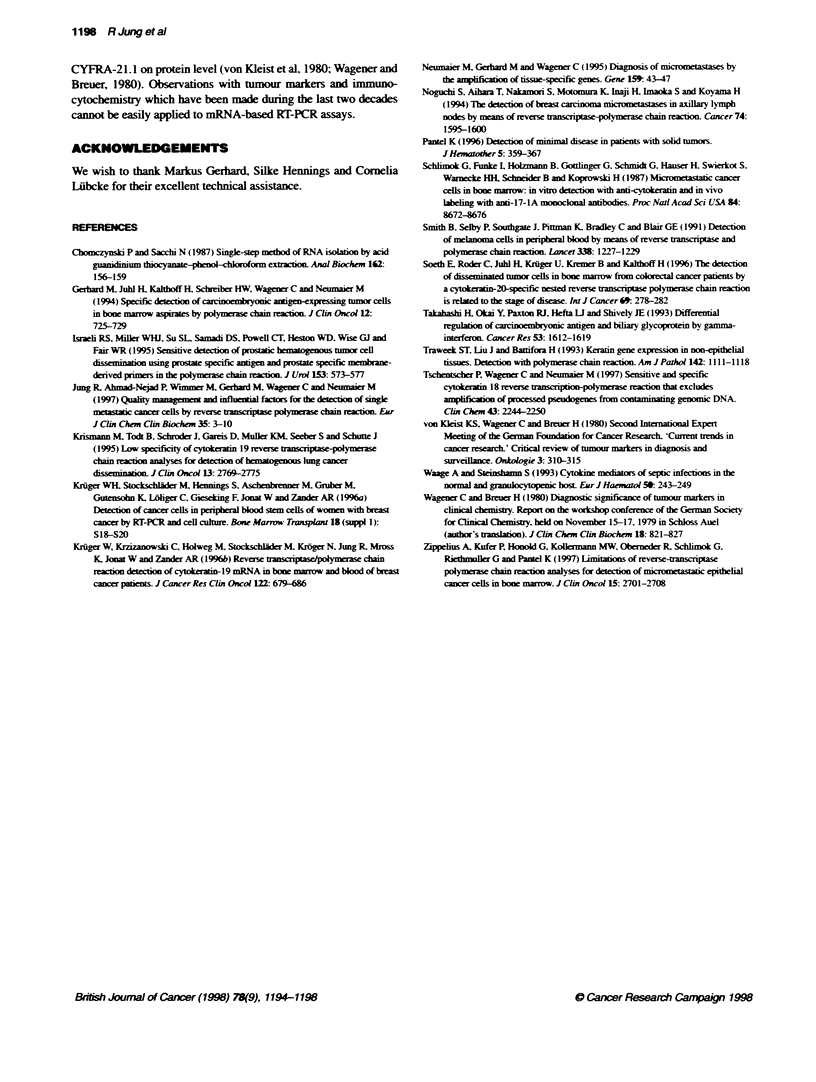# Specificity of reverse transcriptase polymerase chain reaction assays designed for the detection of circulating cancer cells is influenced by cytokines in vivo and in vitro.

**DOI:** 10.1038/bjc.1998.653

**Published:** 1998-11

**Authors:** R. Jung, W. Krüger, S. Hosch, M. Holweg, N. Kröger, K. Gutensohn, C. Wagener, M. Neumaier, A. R. Zander

**Affiliations:** Department of Clinical Chemistry, University Hospital Eppendorf, Hamburg, Germany.

## Abstract

Several reverse transcriptase polymerase chain reaction (RT-PCR) assays have been described for the detection of circulating tumour cells in blood and bone marrow. Target mRNA sequences for this purpose are the cytokeratins (CK) 19 and 20, the carcinoembryonic antigen (CEA), and the prostate-specific antigen messages. In this study, we investigated biological factors influencing the specificity of the CK19 and CEA RT-PCR assays. Bone marrow, granulocyte colony-stimulating factor (G-CSF)-mobilized blood stem cells and peripheral blood samples obtained from healthy volunteers (n = 15; CEA n = 7), from patients with epithelial (n = 29) and haematological (n = 23) cancer and from patients with chronic inflammatory diseases (n = 16) were examined. Neither CEA nor cytokeratin 19 messages could be amplified from bone marrow samples from healthy subjects and from patients with haematological malignancies. In contrast, specimens from patients with inflammatory diseases scored positive up to 60%. To investigate the influence of inflammation on target mRNA expression, haemopoietic cells were cultured with and without cytokine stimulation in vitro. CK19 messages could be easily detected in cultured marrow cells without further stimulation, CEA messages only after gamma-interferon (gamma-INF) stimulation. In contrast, G-CSF-mobilized peripheral blood stem cells were positive for CK19 messages only after stem cell factor (SCF) or interleukin stimulation. We conclude that transcription of so-called tissue-specific genes is inductible in haemopoietic tissues under certain conditions. These factors have to be considered in future applications of RT-PCR for the detection of minimal residual disease.


British Journal of Cancer (1998) 78(9), 1194–1198
© 1998 Cancer Research Campaign

### Specificity of reverse transcriptase polymerase chain reaction assays designed for the detection of circulating cancer cells is influenced by cytokines in vivo and in vitro

**R Jung*[1], W Krüger*[2], S Hosch[3], M Holweg[4], N Kröger[2], K Gutensohn[5], C Wagener[1], M Neumaier[1] and AR Zander[2]**

[1]Department of Clinical Chemistry, [2]Bone Marrow Transplantation Centre, Department of Oncology/Haematology, [3]Department of General Surgery, [5]Department of Transfusion Medicine, University Hospital Eppendorf, 20246 Hamburg, Germany; [4]Department of Gynaecology and Obstetrics, Christian-Albrechts University, 24118 Kiel, Germany

**Summary** Several reverse transcriptase polymerase chain reaction (RT-PCR) assays have been described for the detection of circulating tumour cells in blood and bone marrow. Target mRNA sequences for this purpose are the cytokeratins (CK) 19 and 20, the carcinoembryonic antigen (CEA), and the prostate-specific antigen messages. In this study, we investigated biological factors influencing the specificity of the CK19 and CEA RT-PCR assays. Bone marrow, granulocyte colony-stimulating factor (G-CSF)-mobilized blood stem cells and peripheral blood samples obtained from healthy volunteers ($n = 15$; CEA $n = 7$), from patients with epithelial ($n=29$) and haematological ($n = 23$) cancer and from patients with chronic inflammatory diseases ($n = 16$) were examined. Neither CEA nor cytokeratin 19 messages could be amplified from bone marrow samples from healthy subjects and from patients with haematological malignancies. In contrast, specimens from patients with inflammatory diseases scored positive up to 60%. To investigate the influence of inflammation on target mRNA expression, haemopoietic cells were cultured with and without cytokine stimulation in vitro. CK19 messages could be easily detected in cultured marrow cells without further stimulation, CEA messages only after γ-interferon (γ-INF) stimulation. In contrast, G-CSF-mobilized peripheral blood stem cells were positive for CK19 messages only after stem cell factor (SCF) or interleukin stimulation. We conclude that transcription of so-called tissue-specific genes is inductible in haemopoietic tissues under certain conditions. These factors have to be considered in future applications of RT-PCR for the detection of minimal residual disease.

**Keywords:** CEA; cytokeratin 19; reverse transcriptase polymerase chain reaction; cytokines; micrometastases; epithelial cancer

Currently, reverse transcriptase polymerase chain reaction (RT-PCR) possesses the highest diagnostic sensitivity for the detection of single tumour cells in a variety of tissue specimens and body fluids. Another potentially important application is the quality assessment of leukaphereses products, for example purging of grafts before autologous stem cell transplantation of patients treated with high-dose therapy (Krüger et al, 1996*a*). Eventually, RT-PCR or similar methods will improve staging procedures and, thus, may gain importance for therapeutic management of cancer patients.

Common RNA targets used for these purposes are the carcinoembryonic antigen (CEA) (Gerhard et al, 1994), the cytokeratins 18, 19 and 20 (CK18, CK19, CK20) (Krüger et al, 1996; Tschentscher et al, 1997; Soeth et al, 1996*b*), tyrosinase (Smith et al, 1991) and the prostate-specific antigen (PSA) (Israeli et al, 1995). In contrast to the increasing number of PCR assays published, the gold standard for these applications is currently still the immunocytochemistry (Schlimok et al, 1987; Pantel, 1996). Whereas the reported sensitivity of these RT-PCR assays published is quite similar to one tumour cell in $10^7$ mononucleated blood cells (MNCs), specificity of positive results is discussed very controversially even when the same mRNA targets were used (Krismann et al, 1995; Zippelius et al, 1997). Furthermore, reports vary widely as to the frequency of positive results. To make things even more complex, preanalytical mechanisms which interfere with RT-PCR assays, the lack of standardization, and different assay design or test performance may make a comparison of the reports impossible. The identical sensitivity of the published assays could be attributed to the fact that in those studies the analytical performance is adequate. In addition to the analytical factors that are mentioned above, biological factors influencing RT-PCR results could also be possible. The possibility of induction, alteration, aberrant or low-level expression of target mRNAs under certain conditions has not yet been investigated. However, differences in test specificity might be a result of modified mRNA expression in haemopoietic tissue.

So far, unspecificity attributed to the specific amplification of the tissue-specific expressed mRNA in haemopoietic tissue was explained by accidental pseudogene amplification of pseudogene sequences for the cytokeratins assays or by a general unspecificity of RT-PCR (Neumaier et al, 1995; Zippelius et al, 1997). Most PCR assays have been standardized with samples obtained from patients with epithelial malignancies and from healthy volunteers. Only small collectives of healthy people were used as negative controls and to calibrate and standardize assay sensitivity and specificity. However, no systematic evaluation with patients suffering from non-malignant diseases has been carried out so far.

*Received 11 December 1997*
*Revised 23 March 1998*
*Accepted 16 April 1998*

*Correspondence to:* W Krüger, Bone Marrow Transplantation Centre, Department of Oncology/Haematology, University Hospital Eppendorf, Martinistrasse 52, 20246 Hamburg, Germany

*Equal contributors.

In this study, we compared the CEA and CK19-RT-PCRs by examining bone marrow, peripheral blood and leukapheresis samples obtained from patients with epithelial malignancies ($n = 22$), from patients with non-malignant chronic inflammatory diseases ($n = 16$) and from patients undergoing blood stem cell mobilization and leukapheresis ($n = 20$).

Furthermore, in vitro experiments with cytokine stimulation of haemopoietic cells with and without stroma cells have been carried out to investigate alterations of mRNA expression.

### MATERIAL AND METHODS

### Clinical specimens

We investigated 22 bone marrow samples from patients suffering from different abdominal tumours, and 16 specimens from patients suffering from chronic inflammatory diseases (CID) such as chronic pancreatitis, Crohn's disease and ulcerative colitis. Bone marrow samples from healthy volunteer donors and from patients with haematological malignancies in remission or chronic phase were used as negative controls. Leukapheresis samples were obtained from healthy donors and from patients suffering from different malignancies. Patient samples were received from the Department of Gynaecology and Obstetrics, the Department of Surgery and the Department of Transfusion Medicine. To exclude bias, no detailed information concerning the diagnosis was available to us at the time of analysis.

### Cell lines

For reconstitution experiments and sensitivity testing HT29, MCF7, and MDA-MB453 cells were diluted in normal bone marrow or Buffy coat cells of healthy volunteers between $10^1$ and $10^7$.

### RNA purification

Total RNA was extracted according to standard protocols (Chomczynski and Sacchi, 1987). RNA integrity of each preparation was tested by either β2-microglobulin or β-actin PCR.

### Reverse transcription reaction cDNA synthesis was performed in a 20-μl reaction volume. Ten microlitres of total RNA was used for first strand cDNA synthesis with Superscript II RT (Gibco BRL Life Technologies), according to the manufacturer's recommendations.

### PCR reactions

Sequences of all primers used are shown in Figure 1.

*CEA-PCR*

A total of 20 μl of cDNA was used in the first PCR reaction (PCR I) in a total volume of 50 μl containing 0.5 mM of primers CEAos and CEAoa, 1.5 mM magnesium chloride, 0.1 mM Tris-HCl, 0.04 M ammonium sulphate and 2 U of thermus flavins polymerase (Biozym Diagnostik, Germany). Thirty-five cycles were performed with 1 min at 94°C (denaturing temperature), 1 min at 56°C (annealing temperature) and 1 min at 72°C (extension temperature) (extension time prolonged to 10 min for the last cycle). Nested-PCR was performed using 3 μl of the first PCR as template for primers CEAis, CEAia, β-M1 and β-M2 in 100 μl reaction mix using similar conditions as in PCR I. Thirty-five cycles were performed at 94°C denaturing temperature, 65°C annealing temperature and 72°C extension temperature followed by 10 min at 72°C.

*Cytokeratin 19 PCR*

PCR was carried out as described earlier (Krüger et al, 1996*b*). PCR products were visualized after electrophoresis and ethidium bromide staining on an UV transilluminator. The sensitivity of the CK19 RT-PCR assay was determined to be 1:$10^7$ using dilutions of breast cancer cell line MCF-7 and MDA-MB453 in mononucleated cells of volunteers.

Each sample was investigated twice with both assays and judged as positive if there was at least one positive result.

### Cell culture and cytokine stimulation

Mononucleated cells (MNC) from bone marrow cells harvested from healthy donors and granulocyte colony stimulating factor (G-CSF)-mobilized blood stem cells from patients with haematological malignancies in complete remission were obtained after informed consent. Cells were cultured in RPMI-1640 medium supplemented with 10% fetal calf serum (FCS), L-glutamine, sodium pyruvate and penicillin/streptomycin for 1 week with or without cytokine stimulation respectively. Human recombinant cytokines SCF (5 U ml$^{-1}$), G-CSF (500 U ml$^{-1}$), granulocyte–macrophage colony-stimulating factor (GM-CSF) (500 U ml$^{-1}$),

**Table 1** PCR amplification of cytokeratin 19 message

| Diagnosis | Samples (*n*) | Positive (*n*) | Positive (%) |
|---|---|---|---|
| Healthy marrow donors | 15 | 0 | 0 |
| Haematological malignancies in CR or CP | 23 | 0 | 0 |
| Abdominal cancers | 22 | 11 | 50 |
| CID | 16 | 8 | 50 |
| PBSC (NHL, CML, healthy) | 6 | 0 | 0 |

CR, complete remission; CP, chronic phase; CID, chronic inflammatory disease; PBSC, peripheral blood stem cells; NHL, non-Hodgkin's lymphoma; CML, chronic myeloic leukaemia.

**Table 2** PCR amplification of CEA message

| Diagnosis | Samples (*n*) | Positive (*n*) | Positive (%) |
|---|---|---|---|
| Healthy marrow donors | 7 | 0 | 0 |
| Abdominal cancers | 22 | 13 | 59 |
| CID | 16 | 10 | 62 |
| PBSC (acute leukaemia) | 2 | 2 | 100 |

**Table 3** Correlation of CEA and CK19 RT-PCR assays

| Diagnosis | Consistent results (%) | Inconsistent results (%) |
|---|---|---|
| Abdominal cancers | 14/22 (64) | 8/22 (36) |
| CID | 10/16 (62) | 6/16 (38) |

Table 4 CEA and CK19 mRNA RT-PCR amplification from and immunocytochemical tumour cell (TC) detection in G-CSF-mobilized blood stem cells from stage II and III breast cancer patients

| Patient | CEA RT-PCR | CK19 RT-PCR | TCs/2 × $10^6$ MNCs |
|---|---|---|---|
| 1 | + | + | 0 |
| 1 | + | + | 1 |
| 2 | + | + | 1 |
| 2 | + | + | 0 |
| 3 | + | + | 3 |
| 3 | + | − | 0 |
| 4 | + | + | 2 |
| 4 | + | + | 0 |
| 5 | + | − | 0 |
| 6 | + | − | 0 |
| 7 | + | − | 14 |
| 7 | + | − | 9 |

interleukin 3 (IL-3) (20 U $ml^{-1}$), IL-6 (10 000 U $ml^{-1}$) and γINF (50 U $ml^{-1}$) were added. The negative control was cultured without further stimulation. Cells were fed every other day. MNCs from peripheral blood were cultured as described above with additional phytohaemagglutinin stimulation.

### Immunocytochemistry

Cells (2 × $10^6$) from mononucleated cell fraction after Ficoll separation were spun onto slides using a Shandon cytospin centrifuge. Cytokeratin-positive cells were detected with antibody KL1 (Coulter-immunotech). Labelled cells were detected by the APAAP technique following standard procedures. Breast cancer cell lines MCF-7 and MDA-MB453 were used as positive controls and MNC from non-cancer patients as negative controls. Slides were evaluated by light-microscopy and positive cells were counted (Schlimok et al, 1987: Krüger et al, 1996*a*).

### RESULTS

CEA message as well as CK19 message could not be amplified from marrow samples obtained from healthy volunteer donors (CEA, $n$ = 7; CK19, $n$ = 15) and from marrow samples obtained from patients with haematological malignancies in remission or chronic phase of CML (CK19, $n$ = 23).

After this standardization of both assays, samples from cancer patients were investigated. Marrow samples obtained from patients with abdominal cancers showed for cytokeratin 19 an overall positive rate of 50% (11 out of 22). The same samples

**CEAos:** 5'-GGCCTCTAACCCATGCCCGCAGTAT-'3

**CEAoa:** 5'-AAGCCCAGCTCATTTTTGTATTTTT-'3

**Fragment-size:** 370 bp

**CEAis:** 5'-AGTCTCTGCATCTGGAACTTCTCCTGGT-'3
**CEAia:** 5'-TTTAGACTGTAGCTGTTGCAAATGCTTTAAGG-'3
**Fragment-size:** 172 bp

**K19os:** 5'-TTTGAGACGGAACAGGCTCT-'3
**K19oa:** 5'-CAGCTCAATCTCAAGACCCTG-'3
**Fragment-size:** 426 bp

**K19is:** 5'-GCAGATCGAAGGCCTGAA-'3
**K19ia:** 5'-TGAACCAGGCTTCAGCATC-'3
**Fragment-size:** 209 bp

**β-M1:** 5'-CCTGAATTGCTATGTGTCTGGGTTTGATCCA-'3
**β-M2:** 5'-GGAGCAACCTGCTCAGATACATCAAACATGG-'3
**Fragment-size:** 412 bp

**β-Acos:** 5'-GCGAGAAGATGACCCAGATC-'3
**β-Acoa:** 5'-CCGATCCACACGGAGTACTT-'3
**Fragment-size:** 679 bp

**β-Acis:** 5'-GGACTTCGAGCAAGATATGG-'3
**β-Acia:** 5'-GCAGTGATCTCCTTCTGCATC-'3
**Fragment-size:** 294 bp

**Figure 1** Oligonucleotides used for amplification of CEA, CK19, β-microglobulin and β-actin scored positive in CEA RT-PCR in 59% (13 out of 22), six (27%) patients scored negative in both assays. Concordant results were found in 14 out of 22 (64%) of the samples. Inconsistent results were obtained for eight (36%) patients, three were found to be positive for cytokeratin 19 and five positive for CEA.

As an additional specificity control and to investigate the influence of cell abdominal surgery, marrow samples from patients suffering and undergoing surgical intervention were subjected to both RT-PCR assays. Tables 1–3 show that CEA as well as CK19 messages could be detected by PCR in samples obtained from patients suffering from chronic inflammatory diseases of pancreas and bowel. Positivity rate and consistency of results were similar as for cancer patients.

To investigate the influence of cytokine stimulation on mRNA transcription in vivo and in vitro, G-CSF-mobilized leukaphereses samples were examined. G-CSF-mobilized peripheral blood stem cells from patients without epithelial cancers scored positive by CEA PCR and negative by CK19 RT-PCR (Tables 1 and 2). Additionally, 12 samples of G-CSF-mobilized blood stem cells obtained from seven women with stage II and III breast cancer were examined with both assays. From each specimen, 2 × $10^6$ cells were examined for tumour cells by immunocytochemistry. CEA message was amplified from all samples. The positivity rate

Table 5 PCR amplification of cytokeratin 19 and CEA messages from cultured non-stimulated and cytokine-stimulated leukocytes from healthy bone marrow (BM) and G-CSF-mobilized leukaphereses samples (LP), and peripheral blood (PB). PB was examined after 3 days (d3) of culture because of decreasing cell count

| Sample | d1 | d7 | d7 (SCF) | d7 (G-CSF) | d7 (GM-CSF) | d7 (IL-3) | d7 (IL-6) | d7 (γ-NF) |
|---|---|---|---|---|---|---|---|---|
| CK19 | | | | | | | | |
| BM | − | + | + | + | + | + | + | (+) |
| LP | − | − | + | − | − | +/− | +/− | − |
| CEA | | | | | | | | |
| BM | − | − | − | − | − | − | − | + |
| PB | − | − (d3) | n.d. | n.d. | n.d. | n.d. | n.d. | + (d3) |

© Cancer Research Campaign 1998

for CK19 RT-PCR was 58%; 6 out of 12 (50%) were positive by immunocytochemistry. However, the number of detected tumour cells per sample was very low, with a median of 0.5 (range 0–14) cells per $1.8–2 \times 10^6$ MNCs (Table 4).

Aliquots of bone marrow harvests and leukaphereses samples from healthy subjects and from patients with non-epithelial malignancies were cultured with and without cytokine stimulation for 7 days. Marrow samples converted from negative to positive in the CK19 RT-PCR assay after 7 days of culture with and without further cytokine stimulation. In contrast, specific CEA mRNA could only be amplified from bone marrow after 7 days of γ-interferon (γ-INF) stimulation. Corresponding results for CEA were obtained by stimulation of MNCs derived from peripheral blood with γ-INF. Leukaphereses samples scored positive for CK19 mRNA only after stimulation with SCF, IL-3 and IL-6. According to the results shown in Table 2, examination of CEA expression in stimulated leukaphereses samples was omitted (Table 5).

### DISCUSSION

The discussion in the literature regarding the specificity of RT-PCR assays designed for the detection of occult tumour cells in bone marrow and peripheral blood is very controversial. Suspected reasons for so-called false-positive results of RT-PCR assays are low-level transcription of marker genes in non-epithelial cells, as well as accidental pseudogene amplification. In accordance with our previous reports, no specific amplification of the tissue-specific or epithelial-specific genes in samples of healthy donors was detected (Krüger et al, 1996*b*; Jung et al, 1997). However, in samples obtained from patients suffering from chronic inflammatory diseases, specific but obviously false-positive amplification of both genes was observed quite frequently. Furthermore, for the CEA RT-PCR assay, a positivity rate of 100% in 14 samples of G-CSF-mobilized peripheral stem cells harvested from women suffering from breast cancer indicates that gene expression of so-called tissue-specific antigens may be altered under certain conditions. In vitro studies showed an up-regulation of the CEA message in bone marrow and peripheral blood cells under stimulation with γ-interferon. Stimulation with other cytokines such as IL-3 or IL-6, G-CSF, GM-CSF or SCF as well as cell culture for 7 days did not lead to detectable CEA transcription. Thus, the detected CEA mRNA in materials from CID patients may be induced by cytokines released by inflammation in vivo.

These results are in accordance with data obtained using stimulated HT 29 cells. It is known that γ-interferon and tumour necrosis factor α (TNF-α) lead to an up-regulation of the CEA message in HT 29 cells in vitro and that the CEA gene contains a γ-interferon responsive element (Takahashi et al, 1993). Both cytokines, γ-interferon as well as TNF-α, are involved in the cytokine cascade of acute-phase response (Waage and Steinshamn, 1993). These data suggest that the inflammatory process might induce expression of CEA mRNA in haemopoietic cells. Up-regulation of the CEA transcript by γ-interferon seems to be very specific because neither other cytokines nor global lymphocyte stimulation with phytohaemagglutinin resulted in an increased CEA mRNA expression.

Cytokeratin 19 messages could also be amplified by specific RT-PCR reaction from 50% of marrow samples obtained from patients suffering from inflammatory diseases. The frequent amplification of CK19 messages from marrow samples of patients with Crohn's disease, ulcerative colitis and chronic pancreatitis suggests induction or stimulation of cytokeratin expression in haemopoietic cells by inflammation. However, a direct liberation of CK19 mRNA from dying epithelial cells because of inflammation could be another explanation. To investigate the possibility of CK19 mRNA transcription in haemopoietic tissues, marrow and stem cell samples were cultured with and without the stimulation of several cytokines. CK19 mRNA could be amplified from bone marrow samples after a 7-day culture under standard conditions without additional cytokine stimulation. In contrast, in G-CSF-mobilized peripheral blood stem cells without typical marrow stromal tissue, CK19 mRNA could only be detected after additional stimulation with SCF, IL3, IL6 or γ-INF.

These results lead us to conclude (I) cytokeratin 19 mRNA transcription is easily induced in bone marrow in the presence of stromal cells; (II) that under specific and very artificial conditions cytokeratin transcription is also possible in haemopoietic precursor cells extracted from peripheral blood; (III) the detected specific cytokeratin mRNA in patients with CID may be induced in stromal cells of the reactive marrow by cytokines involved in the inflammatory process. This is in accordance with reports of Traweek et al (1993), who examined the cytokeratin expression in haemopoietic tissue by RT-PCR. CK19 mRNA could not be amplified in this study from mononuclear blood cells, from normal bone marrow or from lymph nodes, but could easily be detected in fibroblasts and endothelial cells under cell culture conditions. Additionally, several groups investigated lymph nodes for micrometastases by cytokeratin 19 RT-PCR and confirmed negativity of non-reactive control nodes (Noguchi et al, 1994).

Thus, it seems that in CID different mechanisms lead to specific but misleading positive results for different target genes because specific amplification is usually judged as the presence of circulating epithelial tumour cells in the specimen. For CEA, a member of the immunoglobulin superfamily, it could be speculated that the mRNA is specifically up-regulated by γ-interferon, whose function remains unclear so far. CK19 mRNA seems to be expressed unspecifically by stimulated stromal cells during the inflammatory process. The meaning of these phenomena is unclear and requires further investigation.

The examination of leukaphereses from patients suffering from stage II and III breast cancer by CK19 RT-PCR and conventional immunocytochemistry gave discordant results. The median tumour cell load per $1.8–2 \times 10^6$ cells was very low with 0.5 per sample. However, only three samples (25%) were negative in both assays. Discordant results were obtained in five (42%) samples. The Poisson distribution of tumour cells in sample aliquots examined by PCR and immunocytochemistry could be responsible for these results. This indicates the necessity to examine clinical samples for contaminating tumour cells by different methods, and, when possible, repeatedly.

We have shown that the pathway to so-called false-positive results obtained by CEA and CK19 RT-PCR assays are completely different. Consequences are (I) both assays are currently not feasible to screen undefined large populations for the presence of tumour cells in bone marrow or peripheral blood; (II) additional markers to discriminate amplification because of inflammatory diseases or cancer should be determined; (III) RT-PCR assays should be combined with immunocytochemistry in further studies to determine the clinical relevance of circulating tumour cells; and (IV) negativity of clinical specimens in repeated PCR examinations indicates a highly probable absence of tumour cells.

These consequences are quite similar to guidelines established for the diagnostic use of tumour marker detection, such as CEA or

© Cancer Research Campaign 1998

CYFRA-21.1 on protein level (von Kleist et al, 1980; Wagener and Breuer, 1980). Observations with tumour markers and immunocytochemistry which have been made during the last two decades cannot be easily applied to mRNA-based RT-PCR assays.

### ACKNOWLEDGEMENTS

We wish to thank Markus Gerhard, Silke Hennings and Cornelia Lübcke for their excellent technical assistance.

### REFERENCES

Chomczynski P and Sacchi N (1987) Single-step method of RNA isolation by acid guanidinium thiocyanate–phenol–chloroform extraction. *Anal Biochem* **162**: 156–159

Gerhard M, Juhl H, Kalthoff H, Schreiber HW, Wagener C and Neumaier M (1994) Specific detection of carcinoembryonic antigen-expressing tumor cells in bone marrow aspirates by polymerase chain reaction. *J Clin Oncol* **12**: 725–729

Israeli RS, Miller WHJ, Su SL, Samadi DS, Powell CT, Heston WD, Wise GJ and Fair WR (1995) Sensitive detection of prostatic hematogenous tumor cell dissemination using prostate specific antigen and prostate specific membrane-derived primers in the polymerase chain reaction. *J Urol* **153**: 573–577

Jung R, Ahmad-Nejad P, Wimmer M, Gerhard M, Wagener C and Neumaier M (1997) Quality management and influential factors for the detection of single metastatic cancer cells by reverse transcriptase polymerase chain reaction. *Eur J Clin Chem Clin Biochem* **35**: 3–10

Krismann M, Todt B, Schroder J, Gareis D, Muller KM, Seeber S and Schutte J (1995) Low specificity of cytokeratin 19 reverse transcriptase-polymerase chain reaction analyses for detection of hematogenous lung cancer dissemination. *J Clin Oncol* **13**: 2769–2775

Krüger WH, Stockschläder M, Hennings S, Aschenbrenner M, Gruber M, Gutensohn K, Löliger C, Gieseking F, Jonat W and Zander AR (1996*a*) Detection of cancer cells in peripheral blood stem cells of women with breast cancer by RT-PCR and cell culture. *Bone Marrow Transplant* **18** (suppl 1): S18–S20

Krüger W, Krzizanowski C, Holweg M, Stockschläder M, Kröger N, Jung R, Mross K, Jonat W and Zander AR (1996*b*) Reverse transcriptase/polymerase chain reaction detection of cytokeratin-19 mRNA in bone marrow and blood of breast cancer patients. *J Cancer Res Clin Oncol* **122**: 679–686

Neumaier M, Gerhard M and Wagener C (1995) Diagnosis of micrometastases by the amplification of tissue-specific genes. *Gene* **159**: 43–47

Noguchi S, Aihara T, Nakamori S, Motomura K, Inaji H, Imaoka S and Koyama H (1994) The detection of breast carcinoma micrometastases in axillary lymph nodes by means of reverse transcriptase-polymerase chain reaction. *Cancer* **74**: 1595–1600

Pantel K (1996) Detection of minimal disease in patients with solid tumors. *J Hematother* **5**: 359–367

Schlimok G, Funke I, Holzmann B, Gottlinger G, Schmidt G, Hauser H, Swierkot S, Warnecke HH, Schneider B and Koprowski H (1987) Micrometastatic cancer cells in bone marrow: in vitro detection with anti-cytokeratin and in vivo labeling with anti-17-1A monoclonal antibodies. *Proc Natl Acad Sci USA* **84**: 8672–8676

Smith B, Selby P, Southgate J, Pittman K, Bradley C and Blair GE (1991) Detection of melanoma cells in peripheral blood by means of reverse transcriptase and polymerase chain reaction. *Lancet* **338**: 1227–1229

Soeth E, Roder C, Juhl H, Krüger U, Kremer B and Kalthoff H (1996) The detection of disseminated tumor cells in bone marrow from colorectal cancer patients by a cytokeratin-20-specific nested reverse transcriptase polymerase chain reaction is related to the stage of disease. *Int J Cancer* **69**: 278–282

Takahashi H, Okai Y, Paxton RJ, Hefta LJ and Shively JE (1993) Differential regulation of carcinoembryonic antigen and biliary glycoprotein by gamma-interferon. *Cancer Res* **53**: 1612–1619

Traweek ST, Liu J and Battifora H (1993) Keratin gene expression in non-epithelial tissues. Detection with polymerase chain reaction. *Am J Pathol* **142**: 1111–1118

Tschentscher P, Wagener C and Neumaier M (1997) Sensitive and specific cytokeratin 18 reverse transcription-polymerase reaction that excludes amplification of processed pseudogenes from contaminating genomic DNA. *Clin Chem* **43**: 2244–2250

von Kleist KS, Wagener C and Breuer H (1980) Second International Expert Meeting of the German Foundation for Cancer Research. 'Current trends in cancer research.' Critical review of tumour markers in diagnosis and surveillance. *Onkologie* **3**: 310–315

Waage A and Steinshamn S (1993) Cytokine mediators of septic infections in the normal and granulocytopenic host. *Eur J Haematol* **50**: 243–249

Wagener C and Breuer H (1980) Diagnostic significance of tumour markers in clinical chemistry. Report on the workshop conference of the German Society for Clinical Chemistry, held on November 15–17, 1979 in Schloss Auel (author's translation). *J Clin Chem Clin Biochem* **18**: 821–827

Zippelius A, Kufer P, Honold G, Kollermann MW, Oberneder R, Schlimok G, Riethmuller G and Pantel K (1997) Limitations of reverse-transcriptase polymerase chain reaction analyses for detection of micrometastatic epithelial cancer cells in bone marrow. *J Clin Oncol* **15**: 2701–2708

© Cancer Research Campaign 1998